# A Novel Lorazepam Orodispersible Film: Bioequivalence, Pharmacokinetic, and Tolerability Evaluation Compared with Film-Coated and Orodispersible Tablets in Two Randomized Crossover Studies in Healthy Subjects

**DOI:** 10.3390/ph19101527

**Published:** 2026-09-26

**Authors:** Valeria Frangione, Cristina Sensi, Milko Massimiliano Radicioni, Fabio Marra, Andrea Maria Giori

**Affiliations:** 1 IBSA Institut Biochimique SA, 6912 Pazzallo, Switzerland; valeria.frangione@ibsa.ch; 2 CROSS Research SA, 6864 Arzo, Switzerland; milko.radicioni@croalliance.com; 3 IBSA Farmaceutici Italia, 26900 Lodi, Italy; fabio.marra@ibsa.it (F.M.);

**Keywords:** lorazepam, bioequivalence, orodispersible film, formulation, pharmacokinetics, treatment adherence

## Abstract

**Introduction:** Lorazepam is a widely prescribed benzodiazepine available in multiple pharmaceutical formulations. A novel lorazepam orodispersible film (ODF) has been developed as an alternative dosage form designed to address administration challenges and enhance treatment adherence. Its pharmacokinetic profile, including the rate and extent of absorption, and its tolerability were evaluated in comparison with commercially available oral formulations in two bioequivalence studies. **Methods:** Two single-center, single-dose, open-label, randomized studies were conducted in healthy adults under fasting conditions. Study A was a three-way crossover pilot study comparing lorazepam 2.5 mg ODF (test, T) with film-coated tablets (Tavor^®^, reference 1, R1) and orodispersible tablets (Tavor^®^ ORO, reference 2, R2). Study B was a two-way crossover pivotal study comparing T with R1. Treatments were separated by washout periods of ≥7 days. Pharmacokinetic parameters and point estimates (PEs), expressed as the ratios of least-squares geometric means (T/R1 and T/R2), were assessed. **Results:** In both studies, lorazepam pharmacokinetic profile was comparable across formulations, with similar C_max_ and area under the curve (AUC) values. Peak concentrations were reached within 1–1.5 h for all treatments, and the elimination half-life was approximately 15–16 h. The 90% CIs for C_max_, AUC_0–t_, and AUC_0–∞_ for T versus R1 and R2 were within the 80–125% bioequivalence range. All treatments were well tolerated, with somnolence being the most frequent adverse event. **Conclusions:** Lorazepam 2.5 mg ODF demonstrated bioequivalence to both film-coated and orodispersible tablet formulations, with a comparable tolerability profile, supporting its potential as a convenient alternative formulation for lorazepam.

## 1. Introduction

Benzodiazepines remain among the most widely prescribed psychotropic medications worldwide, with prevalence estimates approaching 15% among older adults [1]. Although the prevalence of benzodiazepine prescription progressively increases with age, its use among younger adults is not negligible [2,3,4]. Benzodiazepines are classified according to their elimination half-life into short-, intermediate-, and long-acting compounds [5,6,7]. Among the intermediate–short-acting molecules [6,7], lorazepam has an extensive history of clinical use spanning nearly five decades. Like other short-acting benzodiazepines with hypnotic effects, lorazepam is indicated for the short-term management of anxiety disorders, including anxiety-related insomnia [8]. Additionally, it can be used as premedication in anesthesia, for the treatment of status epilepticus, and to manage acute agitation [9,10,11]. In hospital settings, lorazepam is commonly employed due to its rapid onset of action following intravenous administration [10,11,12].

The pharmacological effects of lorazepam are mediated through positive modulation of the γ-aminobutyric acid (GABA)-A receptor complex. By enhancing GABAergic inhibitory neurotransmission in the central nervous system, lorazepam reduces neuronal excitability, leading to its characteristic sedative, muscle relaxant, and amnestic effects [5,7,13].

Lorazepam is available in multiple pharmaceutical forms, including oral tablets, orodispersible tablets (ODTs), liquid oral formulation, and parenteral formulations for intravenous and intramuscular administration. Conventional oral tablets are widely used for routine drug administration; however, they may be associated with swallowing difficulties in certain patient populations, including pediatric, geriatric and psychiatric patients, as well as individuals without immediate access to water or those recovering from anesthesia [14,15,16]. ODTs have been developed to overcome some of these limitations; by rapidly disintegrating in the oral cavity without the need for water, they facilitate administration and reduce swallowing-related difficulties. Nevertheless, evidence that ODTs improve treatment adherence relative to conventional oral formulations remains inconsistent [17]. In addition, ODT development and manufacture present several challenges related to effective taste masking and sufficient mechanical strength [18,19]. Oral liquid formulations represent a valuable alternative and are widely used in pediatric, geriatric, and palliative care populations, providing dosing flexibility and facilitating administration in patients with swallowing difficulties. Yet, the formulation of liquid dosage forms is also complicated by physical and chemical stability concerns. Achieving acceptable taste, smell, and overall palatability is equally important to ensure patient acceptability and adherence [20,21]. Mini-tablets have also emerged as a promising alternative owing to their dosing flexibility and generally superior stability compared with liquid formulations; however, their production requires specialized manufacturing equipment that is not readily available in most compounding pharmacies. As a result, extemporaneously prepared oral liquids remain a key therapeutic option in clinical practice [22].

Orodispersible films (ODFs) have emerged as a promising alternative oral drug delivery platform with unique and advantageous features. According to the European Pharmacopeia, ODFs are thin, single- or multilayer polymeric sheets designed to disintegrate in the oral cavity upon contact with saliva rapidly [23,24]. Similarly to ODTs, ODFs allow administration without the need for water, thereby improving patient compliance, dosing accuracy, and overall portability. In addition, their rapid disintegration and thin, flexible structure facilitate ease of swallowing. Moreover, unlike ODTs, ODFs exhibit improved mechanical properties, including greater flexibility, reduced thickness, increased robustness and superior handling characteristics [18].

Lorazepam has often been employed as a model compound to investigate formulation strategies for oral liquid preparations that are stable, safe, and acceptable to patients [25,26]. In parallel, alternative dosage forms have been explored to overcome the limitations associated with conventional oral formulations. However, the pharmaceutical development of lorazepam-containing formulations is complicated by the molecule’s intrinsic chemical instability. Lorazepam is known to undergo intramolecular dehydration and ring-opening degradation reactions, particularly under acidic conditions and at elevated temperatures [26,27]. Moreover, its limited aqueous solubility necessitates the use of organic co-solvents, such as propylene glycol, polyethylene glycol, and glycerol. Nevertheless, dilution of these formulations in aqueous media, such as 0.9% sodium chloride solution or 5% dextrose solution for intravenous administration, results in a marked reduction in stability [28].

Several studies investigating extemporaneously compounded oral liquid formulations have shown that, although acceptable stability can be achieved under refrigerated conditions, degradation is markedly accelerated at room temperature. Furthermore, formulation-specific factors, including excipient composition, may significantly influence long-term stability [22,29].

These limitations have prompted the search for alternative dosage forms capable of combining ease of administration with improved physicochemical stability. In this context, IBSA Institut Biochimique SA (Lugano-Pazzallo, Switzerland) developed a novel lorazepam ODF formulation, providing an easy-to-administer formulation suitable for emergency situations, episodes of acute agitation, and pre-procedural settings.

The bioequivalence, pharmacokinetic (PK) parameters, as well as the safety and tolerability of the lorazepam ODF formulation were evaluated in two clinical studies. In the preliminary pilot study (22CH-Lrz05, Study A), lorazepam 2.5 mg ODF demonstrated bioequivalence to two commercially available reference products, Tavor^®^ (lorazepam) film-coated tablets and Tavor^®^ (lorazepam) ODTs, when administered under fasting conditions in healthy adults. These findings were subsequently confirmed in the pivotal study (23CH-Lrz06, Study B), which established the bioequivalence of lorazepam 2.5 mg ODF and the Tavor^®^ film-coated tablets under the same conditions in a similar population.

## 2. Results

### 2.1. Study Population

In Study A, 18 healthy subjects (9 males, 9 females; mean age: 39.1 years ± 11.6; mean body weight: 70.31 kg ± 13.76; 88.9% Caucasian) met the eligibility criteria and were randomized and received at least one of the three investigational products. A total of 17 subjects completed the study and were included in the PK analysis set, as one subject withdrew prematurely due to a major protocol deviation (smoking despite prohibition). This subject received only treatment R2 and was therefore included in the safety analysis set for this treatment only.

In Study B, 20 healthy adults (11 males, 9 females, mean age: 41.2 years ± 9.6; mean body weight: 71.23 kg/m^2^ ± 12.69; 95.0% Caucasian) who met the eligibility criteria were enrolled and completed the study. Participant disposition for Studies A and B is shown in Figure 1. Baseline demographics and clinical characteristics of the study populations in Studies A and B are presented in Table 1.

All participants were in good physical and mental health, as confirmed by medical and surgical history assessments and physical examination.

### 2.2. Lorazepam PK Analysis Results

In both Studies A and B, quantifiable plasma concentrations of lorazepam were measured in all subjects from 30 min after single-dose administration of each treatment. In Study A, peak plasma concentrations were reached at 2 h post-dose for T (28.99 ± 6.50 ng/mL), at 1.5 h for R1 (28.72 ± 8.43 ng/mL) and at 1 h for R2 product (31.28 ± 11.92 ng/mL). Similarly, the highest mean concentration was observed at 2 h post-dose after T (24.55 ± 5.99 ng/mL) and at 1 h post-dose after R1 (26.14 ± 8.30 ng/mL) in Study B. Following peak concentrations, plasma lorazepam levels declined quite rapidly and similarly across treatments in both studies; quantifiable concentrations above the lower quantification limit (LQL) were still observed in most subjects at 72 h post-dose. Mean plasma concentration–time profiles of lorazepam in Studies A and B are presented in Figure 2A,B.

In Study A, T, R1 and R2 showed comparable absorption profiles, with similar mean C_max_, AUC_0–t_ and AUC_0–∞_ values. Median T_max_ values were similar across treatments (1.5 h for T and 1 h for both R1 and R2), and t_½_ was approximately 15 h for all treatments (Table 2). Consistent findings were obtained for T and R1 in Study B, where C_max_, AUC_0–t_, and AUC_0–∞_ values were comparable, with no relevant differences in time to peak (median t_max_; 1.5 h after T and 1 h after R1) and half-life (approximately 16 h for both products) (Table 3).

Relative bioavailability was similar across treatments in both studies, with mean F_rel_ values close to 100% in Study A (mean F_rel_ = 102.54% ± 5.02 for T/R1 and mean F_rel_ = 97.40% ± 6.40 for T/R2) and Study B (mean F_rel_ = 104.25 ± 12.76 for T/R1).

Statistical analyses of PK parameters for studies A and B are summarized in Table 2 and Table 3, respectively. In Study A, comparison between T and R1 showed PEs were close to 100% for C_max_, AUC_0–t_, and AUC_0–∞_, and their 90% CIs fell within the predefined bioequivalence acceptance range of 80–125%. No statistically significant difference in t_max_ was observed between T and R1 (*p* = 0.433). Comparable results were obtained for the comparison T vs. R2 (Table 4), including the absence of a significant difference in t_max_ (*p* = 0.424).

In Study B, the comparison T and R showed PEs close to 100%, with 90% CIs inside the 80–125% acceptance interval for both C_max_ and AUC_0–t_ (Table 5). The difference between t_max_ values of the two treatments was not statistically significant (*p* = 0.074).

Once placed in the oral cavity, all orodispersible formulations disintegrated within 2 min, except for one R2 administration in Study A (2 min 44 s) and one T administration in Study B (2 min 59 s). In both studies, T mostly dissolved within approximately 1 min.

### 2.3. Safety Results

Lorazepam ODF and R1 and R2 administered as a single dose were well tolerated by healthy adults in both studies.

In Study A, a total of 61 treatment-emergent adverse events (TEAEs) were reported, with all subjects (100%) experiencing at least one TEAE during the study. The incidence of TEAEs was comparable across treatments, occurring in 88.2% of subjects following T, 94.1% following R1, and 94.4% following R2. The majority of TEAEs (56/61 events) were considered related to study treatment, and all subjects experienced at least one treatment-related TEAE (Table S2).

In Study B, 53 TEAEs were reported across the study population; of these, 27 TEAEs occurred in 19 subjects (95.0%) following T and 26 TEAEs were observed in 20 subjects (100%) following R1. Fifty of the 53 reported TEAEs were considered related to the study treatment, including 26 TEAEs following T in 18 subjects (90%) and 24 TEAEs following R1 in 20 subjects (100%) (Table S3).

All TEAEs were of mild or moderate intensity, with no severe TEAEs or TEAEs leading to discontinuation reported in either study.

Somnolence was the most frequently reported TEAE in both studies, with a comparable incidence across treatments (88.2% with T, 94.1% with R1, and 94.4% with R2 in Study A; 90.0% with T and 100% with R1 in Study B). Other less common TEAEs included: headache (four events in 15% of subjects in Study B, two with T and two with R1), vomiting (four events in 15% of subjects in Study B, three with T and one with R1), nausea (three events in 10% of subjects in Study B, two with T and one with R1), and diplopia (four events in 16.7% of subjects in study A, three with R1 and one with R2; two events in 10% of subjects in Study B, one with T and one with R1).

All events of somnolence, vomiting, nausea, and diplopia and most headache events were considered treatment-related, with the exception of one headache episode requiring paracetamol. In Study B, all TEAEs resolved spontaneously by the end of the study.

No clinically significant effects on vital signs, ECG, body weight, or laboratory parameters were observed; isolated changes in hematology and blood chemistry were not considered related to treatment and were not clinically relevant.

## 3. Discussion

A novel maltodextrin-based ODF formulation of lorazepam was developed and evaluated in two clinical studies. In the preliminary pilot study (Study A), lorazepam 2.5 mg ODF demonstrated bioequivalence to both lorazepam film-coated tablets and ODT formulations when administered under fasting conditions in healthy adults. These findings were subsequently confirmed in the pivotal Study B, which established the bioequivalence of lorazepam 2.5 mg ODF and the film-coated tablet under the same conditions in a similar population.

In both studies, treatments exhibited superimposable PK profiles, with comparable elimination half-lives and no meaningful differences in t_max_. The 90% confidence intervals for C_max_ and AUC_0–t_ (AUC_0–∞_ in Study A) fell within the predefined acceptance range of 80–125%, thus meeting regulatory criteria for bioequivalence. Relative bioavailability (F_rel_) values were close to 100% for both T/R1 and T/R2 in Study A and were confirmed for T/R1 in Study B, further supporting the absence of clinically relevant differences in terms of the rate and extent of lorazepam absorption across formulations.

Overall, the findings are consistent with the well-established PK profile of lorazepam reported in the literature. Following oral administration, lorazepam is rapidly and almost completely absorbed, with peak plasma concentrations typically reached within 2–3 h [8], although the literature reports a broader range (1–6 h), reflecting some degree of interindividual variability [11,30,31,32]. Lorazepam undergoes direct glucuronidation in the liver without involvement of cytochrome P450 enzymes, producing an inactive metabolite that is primarily excreted by the kidneys [9,30,32], with an elimination half-life of less than 24 h (14 ± 5 h) [9]. Collectively, our results demonstrate that lorazepam PK characteristics are preserved with the ODF compared with conventional oral formulations.

Lorazepam tolerability was aligned with the well-known safety profile reported in humans [8]. Somnolence was the most frequent treatment-related TEAE across all treatments in both studies; no severe TEAEs, discontinuations due to TEAEs, nor new safety signals were observed.

The overall incidence and pattern of TEAEs were comparable across formulations, suggesting that the maltodextrin-based ODF does not introduce formulation-related tolerability concerns, in line with previous findings for other ODFs with a similar composition [33,34].

The favorable PK performance observed in both studies is particularly noteworthy, considering the formulation challenges associated with lorazepam, including poor aqueous solubility and limited chemical stability. The ODF technology was specifically developed to address these issues through a carefully optimized excipient composition, resulting in a formulation that combines long-term stability with ease of administration. Notably, although water and lipophilic solvents are generally recognized as potentially destabilizing for lorazepam, the active pharmaceutical ingredient was successfully incorporated into a film manufactured using water as the processing solvent. In addition, maltodextrin, the main excipient of the ODF formulation, is a natural polymer with a well-established safety profile and several favorable properties, including water solubility, neutral taste, good palatability, and cost-effectiveness. Its successful use in formulations containing various active pharmaceutical ingredients supports its suitability for enabling rapid disintegration in saliva while maintaining adequate mechanical strength and formulation stability [35].

The demonstration of bioequivalence observed in both clinical studies therefore suggests that this innovative ODF formulation successfully overcomes the physicochemical limitations traditionally associated with lorazepam while maintaining systemic exposure comparable to that of conventional oral tablets.

The potential clinical advantage of the novel lorazepam ODF formulation lies in its patient-centered mode of administration. Compared with conventional oral dosage forms, liquid or solid, ODFs do not require swallowing or water for administration, and are generally preferred by patients [14,36]. ODFs consist of a thin film that rapidly dissolves in the oral cavity, ensuring prompt drug release and facilitating administration while reducing the risk of choking and aspiration [15,35,37]. Additionally, ODFs offer improved robustness and ease of handling compared to ODTs [5]. These features make ODFs particularly suitable for managing acute conditions. They are highly relevant for lorazepam, which, given its sedative and anxiolytic properties, is frequently used in clinical settings where ease and rapidity of administration are critical [10,18,24]. By eliminating the need for swallowing conventional solid dosage forms and facilitating drug administration, ODFs may contribute to improved treatment acceptance and adherence, especially among individuals who have difficulty taking conventional formulations. Swallowing difficulties are common in specific populations, including pediatric and geriatric patients, as well as individuals with neurological or psychiatric conditions [14,15,24]. While the prevalence of dysphagia is higher among patients with neurological and psychiatric disorders [38,39], it also affects a substantial proportion of the general population (approximately 16–23%), increasing to up to 27% in adults over 76 years of age [39]. Accordingly, difficulty in swallowing tablets and capsules has been reported by one out of 11 primary care patients, with 9.4% showing poor adherence [16]. Against this background, ODFs may represent a valuable delivery system by reducing swallowing-related barriers and improving ease of administration, and potentially enhancing quality of life [14,37]. The expanding number of bioactive compounds formulated as ODFs, including those for treating mental disorders, further underscores the clinical potential of this drug delivery platform [37]. Although lorazepam ODF may offer the potential advantages outlined above, the present studies did not assess patient preference, ease of administration, adherence, clinical effectiveness in acute settings, or quality of life. Accordingly, these considerations should be interpreted as potential clinical benefits of the ODF dosage form, based on its pharmaceutical characteristics and the available literature, rather than as findings of the present studies. Further ad hoc studies are needed to confirm these patient-centered outcomes.

## 4. Materials and Methods

### 4.1. Development and Formulation of Lorazepam ODF

Lorazepam 2.5 mg ODF is a maltodextrin-based ODF formulation containing lorazepam, developed in accordance with patent US12616653B2 “Solid orodispersible pharmaceutical composition in film containing lorazepam” [40].

The final formulation was selected based on the overall performance of the tested prototypes and the robustness of the product characteristics. Systematic compatibility studies performed during the early stages of development confirmed that numerous excipients conventionally employed in oral film technologies promote lorazepam degradation. Incompatible excipients included plasticizers such as glycerol and anhydrous betaine, fillers based on polyvinyl acetate derivatives, and surfactants such as polysorbate 80, macrogolglycerol hydroxystearate, and macrogol-32 stearate. Consequently, a systematic evaluation of alternative excipients was undertaken to identify a formulation capable of ensuring long-term stability [22,25]. This optimization process led to the development of a stable ODF formulation comprising a water-based film-forming system, a plasticizer that is solid at room temperature, and a pharmaceutical-grade oil.

Refined olive oil was selected to replace glycerol as the dispersing agent for lorazepam. Sodium alginate, an anionic polysaccharide, was identified as a suitable alternative to film-forming agents. Sorbitol was selected as the preferred plasticizer, effectively reducing the glass transition temperature of the polymeric matrix, thereby improving film flexibility and processability.

Following excipient selection, the final composition was optimized to achieve an appropriate balance between tensile strength, elasticity, and residual moisture content, all critical parameters for manufacturing performance and long-term physical stability.

Mechanical characterization studies, performed using a texture analyzer in accordance with ASTM D882-18 [41] recommendations, demonstrated that residual moisture content strongly influenced film performance. Within the target moisture range of 11.0–15.0%, the films exhibited ultimate tensile strength values ranging from 1.10 to 5.11 MPa, elongation at break values between 23.4% and 66.7%, and elastic modulus values between 22.8 and 229 MPa, indicating an appropriate balance between flexibility and resistance to handling. Films with moisture levels outside this range became either excessively brittle or overly soft, confirming the importance of moisture control as a critical quality attribute. Detailed mechanical characterization data supporting formulation optimization are provided in Table S1.

Content uniformity testing was performed according to Ph. Eur. 2.9.40 (Uniformity of Dosage Units), using a validated HPLC-UV/DAD method. Individual films were dissolved in water/methanol, appropriately diluted, filtered (0.2 μm), and analyzed on a Waters X-Select C18 column (100 × 4.6 mm, 2.5 μm) using a mobile phase consisting of water/acetonitrile (55:45 *v*/*v*), at a flow rate of 0.6 mL/min and a detection wavelength of 230 nm. Individual units showed lorazepam contents ranging from 97.2% to 99.2% of the nominal dose, with a mean content of 97.8%. The calculated Acceptance Value (AV) was 2.2, complying with the pharmacopoeial acceptance criterion (AV ≤ 15), thereby confirming dose uniformity of the 2.5 mg ODF.

Stability studies were performed in accordance with applicable ICH stability guidance Q1 A. The evaluation included appearance, primary packaging integrity, average weight, lorazepam assay, related substances, dissolution, residual moisture, disintegration time, and microbiological quality. Long-term stability studies performed on three pilot batches demonstrated that all monitored quality attributes remained within predefined specifications throughout 30 months of storage under room-temperature conditions. Lorazepam assay remained between 96.4% and 97.2% of the label claim, dissolution performance was maintained above 94%, disintegration complied with specifications, and no microbiological contamination was detected.

The manufacturing process briefly involves the dissolution of maltodextrin, the plasticizer, the active pharmaceutical ingredient previously dispersed in refined olive oil, and the remaining excipients in water. The resulting formulation is subsequently cast onto a release liner and dried under strictly controlled conditions of temperature, air circulation and coating speed. The dried film is then wound into reels, cut to specifications, punched, and finally individually pouched and sealed into suitable single-dose sachets. The composition and function of excipients are summarized in Table 6.

### 4.2. Materials

In pilot Study A (22CH-Lrz05), three investigational products were evaluated: (a) lorazepam 2.5 mg ODF (IBSA Institut Biochimique SA, Lugano, Switzerland), Test formulation (T); (b) Tavor^®^ 2.5 mg film-coated tablets (Pfizer Italia S.r.l., Latina, Italy), Reference formulation 1 (R1); (c) Tavor^®^ 2.5 mg ODT (Pfizer Italia S.r.l., Latina, Italy), Reference formulation 2 (R2). In pivotal Study B (23CH-Lrz06), T was compared only with R1.

The dose of 2.5 mg of lorazepam was chosen for both studies A and B, as it represents the highest strength available for oral solid formulations in Europe and is commonly used in clinical practice when a higher anxiolytic or sedative effect is required [8]. Therefore, demonstrating bioequivalence at this strength was considered the most appropriate and sensitive approach from both a clinical and regulatory perspective.

The studies were designed in accordance with the European Medicines Agency (EMA) Guideline on the Investigation of Bioequivalence [24]. Based on these guidelines, evaluation at the highest strength is considered sufficient to support a waiver for lower strengths, as all are manufactured using the same process, share an identical qualitative composition, are quantitatively proportional, and differ only in film size. 

### 4.3. Study Design

Study A was a single-center, single-dose, open-label, randomized, three-way crossover pilot bioavailability study conducted between August and October 2022. After screening, subjects received T, R1 and R2 in three study periods, with a washout of at least 7 days between doses.

Study B was a single-center, single-dose, open-label, randomized, two-way crossover pivotal bioavailability study conducted between January and March 2024. After screening, subjects received T and R1 in two study periods, with a washout of at least 7 days between doses.

Both studies were conducted at the Phase I Unit of CROSS Research SA (Arzo, Mendrisio, Switzerland).

After an initial screening period, all treatments were administered orally under fasting conditions (following an overnight fast of at least 10 h) on the morning of Day 1 of each study period. The orodispersible products (T and R2 in Study A, T only in Study B) were administered by placing them directly onto the subject’s tongue; subjects were allowed to complete disintegration in the oral cavity without chewing or swallowing and without water. R1 was swallowed intact with water and not chewed. Administration times were recorded for all treatments; dissolution times in the mouth were registered for T (in both studies A and B) and R2 (in Study A).

Due to the known sedative effects of lorazepam [8], subjects were confined to the Phase I Unit from the evening of Day −1 until 72 h post-dose (Day 4) during each study period in both studies. During confinement, standardized meals and water were provided, with no additional food or beverages permitted.

The studies were conducted following the Declaration of Helsinki and ICH-GCP principles. They were both approved by an independent Ethics Committee (Comitato Etico Cantonale, Canton Ticino, Study A Project ID: 2022-00923—CE 4109; Study B Project ID: 2023-01937—CE4476) and by Swissmedic (Study A reference number: 701372; Study B reference number: 701745).

### 4.4. Study Population and Randomization

Both studies enrolled healthy adult males and females. Eligibility was determined based on the following inclusion criteria: provision of written informed consent prior to participation; age between 18 and 55 years; body mass index (BMI) ranging from 18.5 and 30 kg/m^2^; systolic blood pressure between 100 and 139 mmHg, diastolic blood pressure between 50 and 89 mmHg, and resting heart rate of 50–90 bpm in Study A and 50–99 bpm in Study B; ability to understand the nature and purpose of the study; and for women of childbearing potential, use of at least one reliable method of contraception. Subjects were excluded if they presented with: clinically significant abnormalities in electrocardiogram (ECG), physical examination, or laboratory parameters; known hypersensitivity to the active substance or any excipients; a relevant history of diseases that could interfere with study outcomes; use of medications or herbal products within 2 weeks prior to study start; history of drug, alcohol, or tobacco abuse; abnormal dietary habits; pregnancy; or blood donations for 3 months before this study. An additional exclusion criterion in Study B was a positive COVID-19 rapid test result on Day −1.

In Study A, eligible subjects were randomized to one of six treatment sequences (T/R1/R2, R1/T/R2, R2/T/R1, R2/R1/T, T/R2/R1, or R1/R2/T) administered over three periods. In Study B, eligible subjects were randomized to receive one of the two treatments (either T or R1) during period 1 and the other treatment during period 2. Randomization schedules were computer-generated using the PLAN procedure in SAS^®^ version 9.3 (TS1M1).

### 4.5. PK Analysis

Sample collection and PK analysis were conducted according to the same protocol in both studies A and B.

Venous blood samples for PK analysis were collected using an indwelling catheter with a switch valve and collected into 6 mL EDTA tubes by means of a Vacutainer blood transfer device.

Blood samples were collected at the following times in each study period: pre-dose (0 min), 0.5 h (30 min), 1 h, 1.5, 2, 2.5, 3, 3.5, 4, 5, 6, 8, 12, 16, 24, 36, 48 and 72 h post-dose. After collection, blood samples were stored on ice for a maximum of 60 min and then centrifuged at 4° C for 10 min at 2000 ± 20 g to separate plasma, which was aliquoted in polypropylene tubes and stored at ≤−20° C until analysis.

Lorazepam plasma concentration was determined at Syneos Health Clinique Inc., Quebec city, QC, Canada, using a fully validated liquid chromatography–tandem mass spectrometry (LC-MS/MS) method with a lower quantification limit (LQL) of 0.40 ng/mL (range 0.40–80 ng/mL). Analyses were performed according to the general Principles of “OECD Good Laboratory Practices for testing of chemicals” C(81) 30 (final) and applicable GCP regulations. The method demonstrated excellent specificity, linearity over the intended concentration range (R^2^ ≥ 0.9955), accuracy, and precision, complying with predefined validation acceptance criteria. Evaluated PK parameters included: maximum plasma concentration (C_max_), area under the concentration–time curve from administration to the last observed concentration time (AUC_0–t_), area under the concentration–time curve extrapolated to infinity (AUC_0–∞_), time to achieve C_max_ (t_max_), terminal elimination rate constant (λ_z_), half-life (t_1/2_), relative bioavailability (F_rel_). F_rel_ was calculated as the ratio AUC_0–t_ (T)/AUC_0–t_ (R1) and the ratio AUC_0–t_ (T)/AUC_0–t_ (R2). The full set of PK parameters was measured and/or calculated using the validated software Phoenix WinNonlin^®^ version 8.3.5 (Certara USA, Inc., Princeton, NJ, USA).

### 4.6. Endpoints

In Study A, the primary endpoints were the rate (C_max_) and extent (AUC_0–t_ and AUC_0–∞_, if feasible) of lorazepam absorption in plasma after single doses of T, R1 and R2, under fasting conditions. Similarly, in Study B, the primary endpoint comprised C_max_ and AUC_0–t_ of single doses of T and R1, under fasting conditions. In both studies, secondary endpoints included additional PK parameters (t_max_, λz, t½, F_rel_) to further characterize the PK profiles of lorazepam formulations, as well as the collection of safety data.

### 4.7. Safety

Safety and general tolerability of T and reference products were assessed based on the recording of TEAEs and vital sign measurements throughout the study. These included blood pressure, heart rate (at screening, Day −1, Days 1–4 at pre-dose, 1.5, 3, 24, 48 and 72 h post-dose and final visit), ECG (at screening and final visit only), physical examination and laboratory analyses (blood and urine samples for routine hematology, clinical chemistry, virology, and urinalysis at screening and final visit only).

### 4.8. Statistical Analyses

A total of 18 healthy men and women were required to ensure at least 12 subjects completed Study A. No formal sample size calculation was performed; the planned sample size was estimated as sufficient for the exploratory purposes of this pilot study.

For Study B, the sample size calculation was determined using intrasubject variability estimates from the pilot study and standard bioequivalence criteria (α = 0.05, 80% power) for AUC_0–t_ and C_max_. A minimum of 16 subjects was required to ensure adequate power, and 20 subjects were enrolled to account for possible dropouts.

In both studies, descriptive statistics were used to summarize quantitative variables, while qualitative variables were described using frequency distributions. The statistical analyses on PK parameters were performed using SAS^®^ (version 9.3, TS1M1) and Phoenix WinNonlin^®^ (version 8.3.5).

PK parameters AUC_0–t_, AUC_0–∞_ (if feasible) and C_max_ were compared between T and R1 and between T and R2 using analysis of variance (ANOVA) on log-transformed (natural logarithm) data. The statistical analysis included treatment, period, sequence, and subject within sequence as fixed effects. Point estimates (PEs), expressed as the ratios of least-squares geometric means (T/R1 and T/R2), and their corresponding 90% confidence intervals (CIs) were calculated. Bioequivalence was confirmed if the 90% CIs for these ratios fell within the predefined acceptance range of 80–125%, in accordance with the EMA guideline on the investigation of bioequivalence (CPMP/EWP/QWP/1401/98 Rev. 1/Corr**, 2010). Comparisons of T_max_ between treatments (T vs. R1 and T vs. R2) were performed using the Wilcoxon signed-rank test.

## 5. Strengths and Limitations

These studies have several strengths. The randomized crossover design minimized intersubject variability and enabled robust comparisons across formulations. While Study B formally demonstrated the bioequivalence of lorazepam ODF to film-coated tablets, Study A provided a more comprehensive assessment of ODF performance by including comparisons with both film-coated tablets and ODTs. The studies were conducted under controlled conditions in accordance with regulatory guidelines, ensuring high data quality and reliability. Some limitations should also be acknowledged. First, the studies enrolled a relatively small number of healthy volunteers; therefore, the findings may not be fully generalizable to patient populations, including older adults and individuals with psychiatric, neurological, or swallowing disorders. Second, the single-dose design did not permit evaluation of PK, safety, or tolerability during repeated administration or long-term use. Third, the studies were conducted only under fasting conditions and therefore did not assess the potential impact of food on lorazepam bioavailability. Finally, patient acceptability, preference, ease of administration, adherence, and clinical effectiveness were not evaluated. The studies were conducted in a relatively small number of healthy volunteers, which may limit the generalizability of the findings. Furthermore, the evaluation was performed under fasting conditions only and therefore does not account for potential food effects.

## 6. Conclusions

Lorazepam 2.5 mg ODF demonstrated bioequivalence and comparable tolerability to reference oral formulations. Similar PK and safety profiles, together with the absence of expected differences in clinical efficacy, support the use of ODF as an alternative oral formulation for lorazepam, which may offer a more favorable patient-centered option in situations where conventional dosage forms are less convenient or acceptable. Future studies evaluating preference, ease of use, and treatment adherence in target patient populations are warranted to determine whether the use of lorazepam ODF confers meaningful clinical benefits in clinical practice.

## Figures and Tables

**Figure 1:**
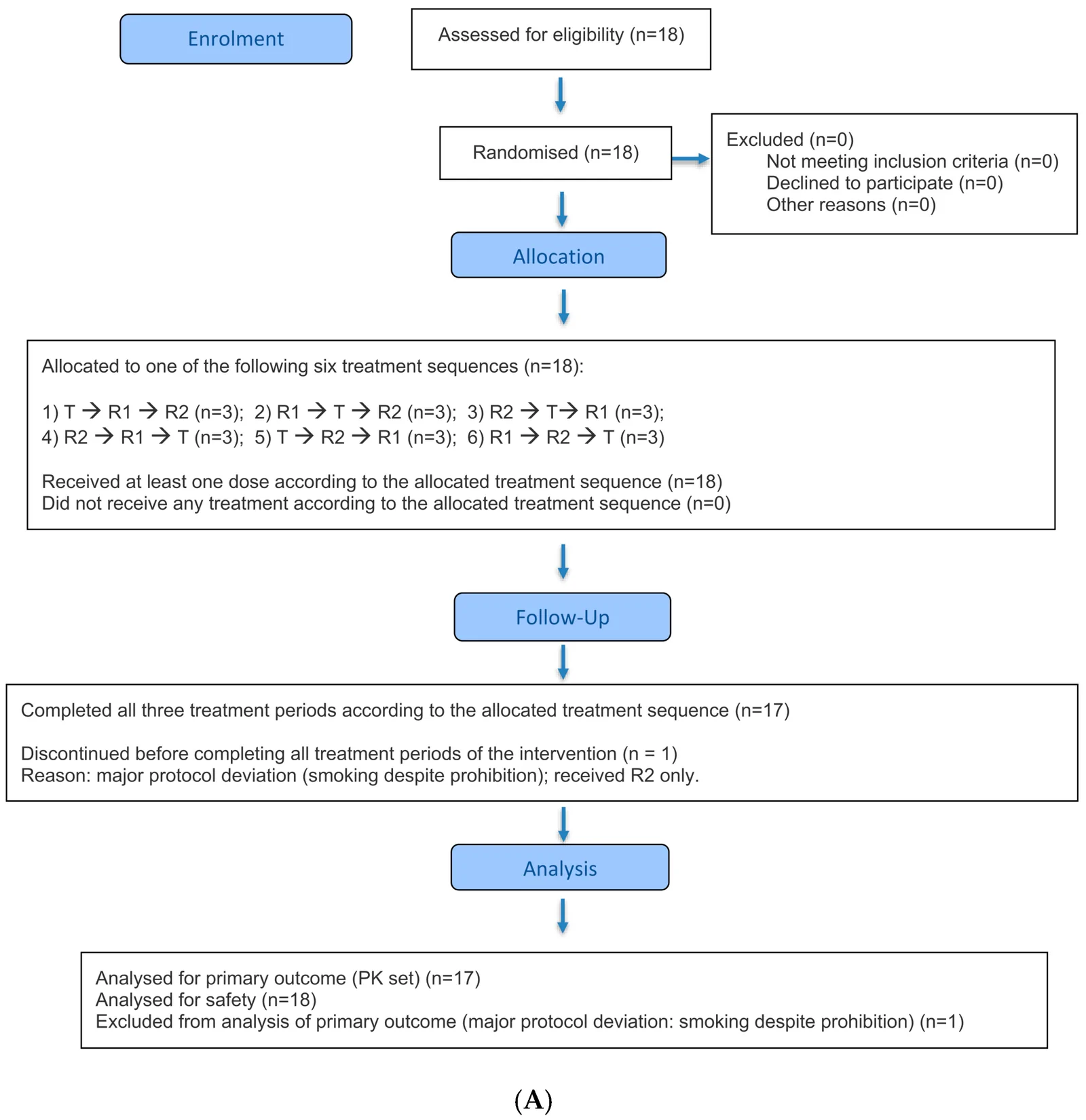

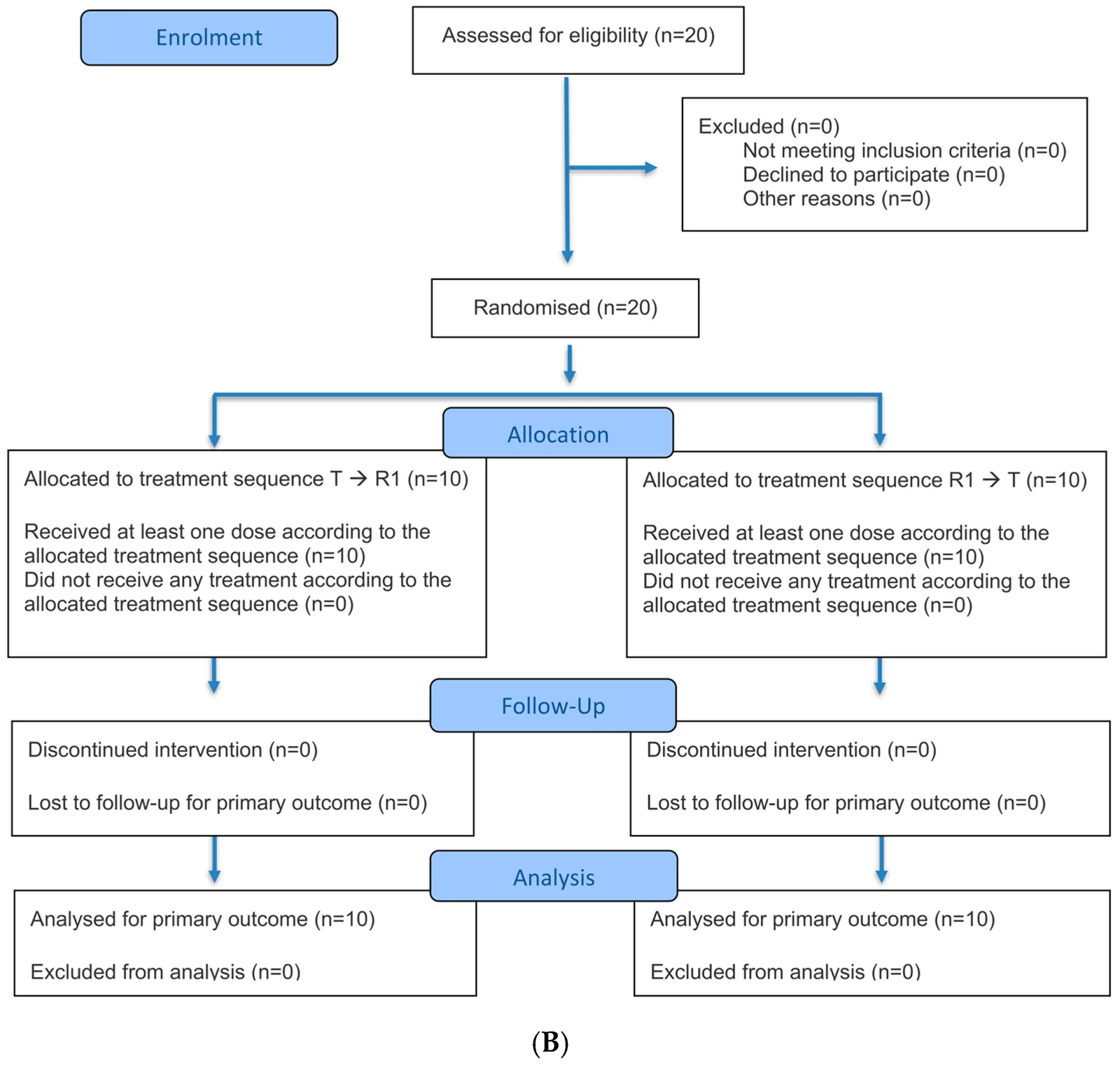
CONSORT 2025 flow diagrams of participant disposition in Studies A and B. Participant progress through enrolment, treatment allocation, follow-up, and data analysis is shown for Study A (22CH-Lrz05) (**A**) and Study B (23CH-Lrz06) (**B**). T: Lorazepam 2.5 mg ODF (IBSA); R1: lorazepam 2.5 mg film-coated tablets (Tavor^®^, Pfizer Italia S.r.l., Latina, Italy); R2: lorazepam 2.5 mg ODT (Tavor^®^, Pfizer Italia S.r.l., Latina, Italy).

**Figure 2:**
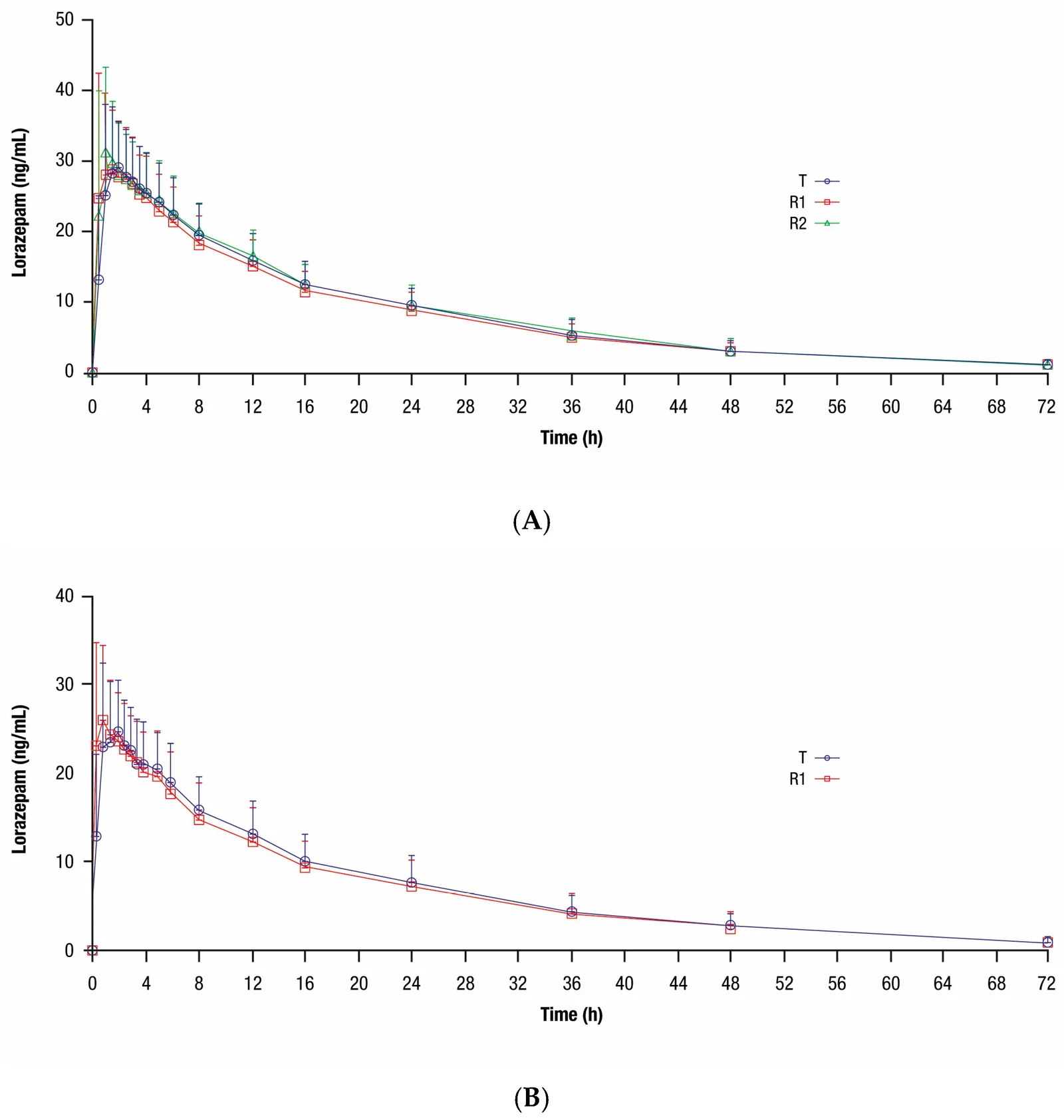
Comparative pharmacokinetic profiles of lorazepam formulations following a single 2.5 mg dose in Studies A and B. (**A**) Mean plasma concentration-time profiles of lorazepam following a single 2.5 mg dose of T, R1, and R2 (Study A). (**B**) Mean plasma concentration-time profiles of lorazepam following a single 2.5 mg dose of T and R1 (Study B). T: Lorazepam 2.5 mg ODF (IBSA); R1: lorazepam 2.5 mg film-coated tablets (Tavor^®^, Pfizer Italia S.r.l., Latina, Italy); R2: lorazepam 2.5 mg ODT (Tavor^®^, Pfizer Italia S.r.l., Latina, Italy).

**Table 1:** Baseline characteristics of participants in Study A and Study B.

**Study A**		
**Characteristics**	**Safety Set (** ***n* = 18)**	**PK Set (** ***n* = 17)**
Sex		
Female, *n* (%)	9 (50%)	9 (52.9%)
Male, *n* (%)	9 (50%)	8 (47.1%)
Age (years)		
Mean ± SD	39.1 ± 11.6	38.3 ± 11.5
Body weight (kg)		
Mean ± SD	70.31 ± 13.76	70.84 ± 13.99
Height (cm)		
Mean ± SD	169.9 ± 9.6	170.1 ± 9.9
BMI (kg/m^2^)		
Mean ± SD	24.14 ± 2.95	24.29 ± 2.97
Ethnic background		
White, *n* (%)	16 (88.9%)	15 (88.2%)
Black or African-American, *n* (%)	1 (5.6%)	1 (5.9%)
Other, *n* (%)	1 (5.6%)	1 (5.9%)
**Study B**		
**Characteristics**	**Safety Set (** ***n* = 20)**	**PK Set (*n* = 20)**
Sex		
Female, *n* (%)	9 (45%)	9 (45%)
Male, *n* (%)	11 (55%)	11 (55%)
Age (years)		
Mean ± SD	41.2 ± 9.6	41.2 ± 9.6
Body weight (kg)		
Mean ± SD	71.23 ± 12.69	71.23 ± 12.69
Height (cm)		
Mean ± SD	172.3 ± 9.0	172.3 ± 9.0
BMI (kg/m^2^)		
Mean ± SD	23.88 ± 3.13	23.88 ± 3.13
Ethnic background		
White, *n* (%)	19 (95.0%)	19 (95.0%)
Other, *n* (%)	1 (5.0%)	1 (5.0%)

PK: pharmacokinetics; SD: standard deviation.

**Table 2:** Lorazepam plasma PK parameters in Study A (PK set, *n* = 17).

PK Parameters	T (Means ± SD)	R1 (Means ± SD)	R2
C_max_ (ng/mL)	32.90 ± 8.47	35.29 ± 12.68	34.48 ± 11.12
AUC_0–t_ (ng/mL × h)	589.62 ± 159.62	573.17 ± 144.27	607.48 ± 166.13
AUC_0–∞_ (ng/mL × h)	615.62 ± 176.59	599.12 ± 158.48	636.49 ± 186.05
t_max_ (h), median (min–max)	1.5 (0.5–3.5)	1.0 (0.5–3.5)	1.0 (0.5–3)
t_½_ (h)	15.15 ± 2.13	15.60 ± 1.80	15.48 ± 2.40
λ_z_ (1/h)	0.05 ± 0.01	0.05 ± 0.01	0.05 ± 0.01

T: Lorazepam 2.5 mg ODF (IBSA); R1: Tavor^®^ 2.5 mg film-coated tablets (lorazepam, Pfizer Italia S.r.l., Latina, Italy); R2: Tavor^®^ 2.5 mg ODT (lorazepam, Pfizer Italia S.r.l., Latina, Italy); AUC: area under the curve; h: hours; PK: pharmacokinetics.

**Table 3:** Lorazepam plasma PK parameters in Study B (PK set, *n* = 20).

PK Parameters	T (Means ± SD)	R1 (Means ± SD)
C_max_ (ng/mL)	28.12 ± 6.18	30.00 ± 8.10
AUC_0–t_ (ng/mL × h)	492.52 ± 153.39	478.95 ± 163.62
AUC_0–∞_ (ng/mL × h)	521.24 ± 173.22	507.25 ± 187.11
t_max_ (h), median (min–max)	1.50 (1–5)	1.00 (0.5–3)
t_½_ (h)	16.25 ± 3.09	15.99 ± 2.88
λ_z_ (1/h)	0.05 ± 0.01	0.05 ± 0.01

T: Lorazepam 2.5 mg ODF (IBSA); R1: Tavor^®^ 2.5 mg film-coated tablets (lorazepam, Pfizer Italia S.r.l., Latina, Italy); AUC: area under the curve; h: hours; PK: pharmacokinetics.

**Table 4:** Statistical comparison of lorazepam PK parameters between T and R1, and between T and R2 (Study A, PK set, *n* = 17).

Treatment Comparison	Parameter	PE (%)	90% CI
T vs. R1	C_max_	95.32	85.53–106.23
	AUC_0–t_	102.54	100.66–104.45
	AUC_0–∞_	102.37	100.31–104.46
T vs. R2	C_max_	96.97	88.51–106.23
	AUC_0–t_	97.34	94.59–100.17
	AUC_0–∞_	97.08	93.91–100.35

T: Lorazepam 2.5 mg ODF (IBSA); R1: Tavor^®^ 2.5 mg film-coated tablets (lorazepam, Pfizer Italia S.r.l., Latina, Italy); R2: Tavor^®^ 2.5 mg ODT (lorazepam, Pfizer Italia S.r.l., Latina, Italy); AUC: area under the curve; CI: confidence interval; PE: point estimate; PK: pharmacokinetics.

**Table 5:** Statistical comparison of lorazepam PK parameters between T and R1 (Study B, PK set, *n* = 20).

Treatment Comparison	Parameter	PE (%)	90% CI
T vs. R1 (Study B)	C_max_	94.63	87.55–102.27
	AUC_0–t_	103.62	99.38–108.05

T: Lorazepam 2.5 mg ODF (IBSA); R1: Tavor^®^ 2.5 mg film-coated tablets (lorazepam, Pfizer Italia S.r.l., Latina, Italy). AUC: area under the curve; CI: confidence interval; PE: point estimate; PK: pharmacokinetics.

**Table 6:** Composition of T (lorazepam 2.5 mg ODF, IBSA) and description of excipient functions.

Ingredient	Function
Lorazepam	Active principle
Maltodextrin	Film-forming polymer
Sorbitol liquid non-crystallizing	Plasticizer and sweetening agent
Purified water	Residual solvent/plasticizer
Sodium alginate	Film-forming polymer
Olive oil, refined	Dispersing agent
Oleoyl macrogol-6 glycerides	Surfactant
Polyglyceryl-3 dioleate	Surfactant
Sucralose	Sweetener
Brilliant Blue FCF	Coloring agent
Titanium dioxide *	Coloring agent

* Present only in the test formulation of Study A and subsequently removed from the Study B formulation following the EFSA Panel on Food Additives and Flavourings (FAF) updated safety assessment of titanium dioxide (E171) [42].

## Data Availability

The datasets generated and analyzed during the current studies are not publicly available. These data are protected by a confidentiality agreement with the study sponsor, IBSA Institut Biochimique SA, due to their ethically and commercially sensitive nature. Data are available from the corresponding author on reasonable request.

## Supplementary Materials

The following supporting information can be downloaded at https://www.mdpi.com/article/10.3390/ph19101527/s1, Table S1: Effect of residual moisture content on mechanical properties of lorazepam ODFs; Table S2: Summary of TEAEs in Study A (safety set, *n* = 18); Table S3: Summary of TEAEs in Study B (safety set, *n* = 20).

## Abbreviations

The following abbreviations are used in this manuscript:

AUC	Area under the curve
AV	Acceptance value
CI	Confidence interval
FAF	Food Additives and Flavourings
GABA	γ-Aminobutyric acid
NA	Not applicable
ODF	Orodispersible film
ODT	Orodispersible tablet
PE	Point estimate
PK	Pharmacokinetic
SD	Standard deviation
TEAE	Treatment-emergent adverse event
