# Supplementary material for: A Novel Lorazepam Orodispersible Film: Bioequivalence, Pharmacokinetic, and Tolerability Evaluation Compared with Film-Coated and Orodispersible Tablets in Two Randomized Crossover Studies in Healthy Subjects
Source: Pharmaceuticals (Basel). 2026 Sep 26;19(10):1527. doi: 10.3390/ph19101527 (PMC13708311; doi:10.3390/ph19101527)
Supplement: Supplementary file 1 [file pharmaceuticals-19-01527-s001.zip › pharmaceuticals-4469967-supplementary.pdf]

Article

A Novel Lorazepam Orodispersible Film: Bioequivalence, Pharmacokinetic, and Tolerability Evaluation Compared with Film-Coated and Orodispersible Tablets in Two Randomized Crossover Studies in Healthy Subjects

Valeria Frangione [1], Cristina Sensi [1,*], Milko Massimiliano Radicioni [2], Fabio Marra [3], Andrea Maria Giori [3]

Supplementary material

Table S1. Effect of residual moisture content on mechanical properties of lorazepam ODFs.

Batch	Moisture content (%)	Tensile properties				
		Thickness (µm)	Force (N)	Ultimate Tensile Strenght (MPa)	Elongation at break (%)	Elastic modulus (MPa)
**5L13HA**	9.8	105 ± 8	24.3±1.80	7.81±0.988	10.4±1.52	368±37.6
**5L13GA**	11.1	113 ± 3	17.2±0.924	5.11±0.380	23.4±3.58	229±31.6
**5L13FA**	11.6	110 ± 3	15.0±0.478	4.57±0.106	27.6±1.15	199±12.2
**5L13AA**	13.1	118 ± 3	7.86±0.567	2.22±0.198	36.5±4.98	82.6±15.6
**5L13BA**	14.0	123 ± 2	4.07±0.452	1.10±0.140	66.7±8.01	22.8±3.79
**5L13CA**	15.7	127 ± 3	3.15±0.431	0.831±0.131	64.2±1.87	13.8±5.51
**5L13DA**	16.9	129 ± 2	2.47±0.470	0.638±0.121	61.0±9.78	10.7±2.28
**5L13EA**	18.0	136 ± 4	1.36±0.0448	0.333±0.0098	72.1±15.9	3.0±0.975

ODF: orodispersible film

Table S2. Summary of TEAEs in Study A (safety set, n=18).

Category	T (n=17)		R1 (n=17)		R2 (n=18)		Overall (n=18)	
	TEAE (n)	Subjects, n (%)	TEAE (n)	Subjects, n (%)	TEAE (n)	Subjects, n (%)	TEAE (n)	Subjects, n (%)
**All TEAEs**	18	15 (88.2)	22	16 (94.1)	21	17 (94.4)	61	18 (100)
**Related**	17	15 (88.2)	19	16 (94.1)	20	17 (94.4)	56	18 (100)
**Not related**	1	1 (5.9)	3	1 (5.9)	1	1 (5.6)	5	3 (16.7)
**Severe**	0	0 (0.0)	0	0 (0.0)	0	0 (0.0)	0	0 (0.0)
**Leading to discontinuation**	0	0 (0.0)	0	0 (0.0)	0	0 (0.0)	0	0 (0.0)

T: Lorazepam 2.5 mg ODF (IBSA); R1: Tavor® 2.5 mg film-coated tablets (lorazepam, Pfizer Italia S.r.l., Italy); R2: Tavor® 2.5 mg ODT (lorazepam, Pfizer Italia S.r.l., Italy); TEAE: treatment-emergent adverse event.

Table S3. Summary of TEAEs in Study B (safety set, n=20).

Category	T (n=20)		R1 (n=20)		Overall (n=20)	
	TEAE (n)	Subjects, n (%)	TEAE (n)	Subjects, n (%)	TEAE (n)	Subjects, n (%)
**All TEAEs**	27	19 (95.0)	26	20 (100)	53	20 (100)
**Related**	26	18 (90.0)	24	20 (100)	50	20 (100)
**Not related**	1	1 (5.0)	2	2 (10.0)	3	2 (10.0)
**Severe**	0	0 (0.0)	0	0	0	0 (0.0)
**Leading to discontinuation**	0	0 (0.0)	0	0	0	0 (0.0)

T: Lorazepam 2.5 mg ODF (IBSA); R1: Tavor® 2.5 mg film-coated tablets (lorazepam, Pfizer Italia S.r.l., Italy); TEAE: treatment-emergent adverse event.
